# Immunosuppressive Agents—Effects on the Cardiovascular System and Selected Metabolic Aspects: A Review

**DOI:** 10.3390/jcm12216935

**Published:** 2023-11-05

**Authors:** Bianka Opałka, Michał Żołnierczuk, Marta Grabowska

**Affiliations:** 1 Department of Histology and Developmental Biology, Faculty of Health Sciences, Pomeranian Medical University, 71-210 Szczecin, Poland; bianka.opalka@onet.pl; 2 Department of Plastic, Endocrine and General Surgery, Pomeranian Medical University, 72-010 Szczecin, Poland; mzolnierczuk98@gmail.com

**Keywords:** immunosuppressive therapy, side effects, cardiovascular system

## Abstract

The widespread use of immunosuppressive drugs makes it possible to reduce inflammation in autoimmune diseases, as well as prevent transplant rejection in organ recipients. Despite their key action in blocking the body’s immune response, these drugs have many side effects. These actions primarily affect the cardiovascular system, and the incidence of complications in patients using immunosuppressive drugs is significant, being associated with a higher incidence of cardiovascular incidents such as myocardial infarction and stroke. This paper analyzes the mechanisms of action of commonly used immunosuppressive drugs and their impact on the cardiovascular system. The adverse effect of immunosuppressive drugs is associated with toxicity within the cardiovascular system, which may be a problem in the clinical management of patients after transplantation. Immunosuppressants act on the cardiovascular system in a variety of ways, including fibrosis and myocardial remodeling, endothelium disfunction, hypertension, atherosclerosis, dyslipidemia or hyperglycaemia, metabolic syndrome, and hyperuricemia. The use of multidrug protocols makes it possible to develop regimens that can reduce the incidence of cardiovascular events. A better understanding of their mechanism of action and the range of complications could enable physicians to select the appropriate therapy for a given patient, as well as to reduce complications and prolong life.

## 1. Introduction

Advances in immunosuppressive therapy have led to the development of treatment protocols using combinations of different drugs that effectively reduce the number of acute rejection episodes. For transplant recipients, immunosuppressive protocols are usually based on combinations of three drugs from different classes that provide different mechanisms of action. Conventional immunosuppressive protocols used in clinical practice include cyclosporin A (CsA), tacrolimus (FK-506), sirolimus/rapamycin (RAP), mycophenolate mofetil (MMF), azathioprine (AZA), and glucocorticoids (GCS). New drugs currently used in transplantation include basiliximab (BAS) and anti-thymocyte globulin (ATG) (Table 1). Each of these drugs exhibits different mechanisms of action on the activation cascade and T-cell proliferation. The wide spectrum of applications for these drugs allows for many therapeutic possibilities [1,2].

However, in patients who have undergone organ transplantation, attention is drawn to the increased cardiovascular risk resulting from the use of immunosuppressive drugs, as well as to the many risk factors associated with organ transplantation itself. Knowledge of these factors is important to better tailor the therapy to a particular patient and to take into account the overall cardiovascular risk to reduce the incidence of complications and mortality in patients taking immunosuppressive drugs. To better understand immunosuppressive drugs, it is necessary to analyze their different mechanisms of action, as well as the risk factors associated with their intake that predispose patients to cardiovascular disease (CVD).

## 2. Short Characteristics of Selected Immunosuppressive Drugs

### 2.1. Cyclosporin A

**Figure 1:**
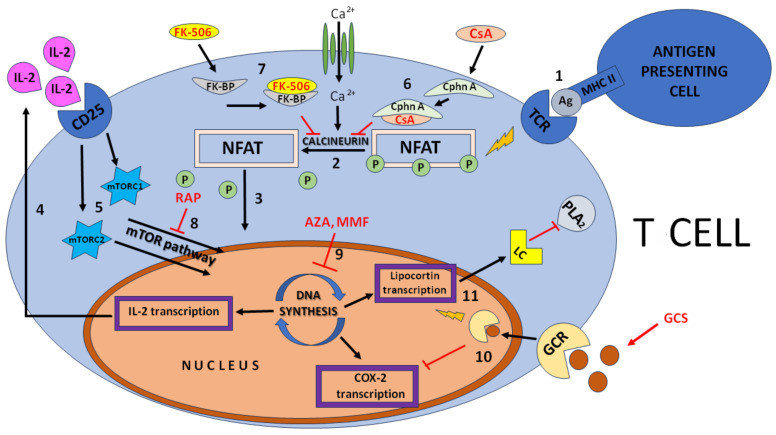
Simplified mechanism of action of immunosuppressive drugs. MHC II—major histocompatibility complex class II; Ag—antigen; TCR—T-cell receptor; P—phosphate; NFAT—nuclear factor of activated T cells; FK-BP—FK506-binding protein; Cphn A—cyclophilin A; IL-2—interleukin 2; mTOR—mammalian target of rapamycin; LC—lipocortin; PLA_2_—phospholipase A_2_; GCR—glucocorticoid receptor; GCSs—glucocorticoids; AZA—azathioprine; MMF—mycophenolate mofetil; RAP—rapamycin; FK-506—tacrolimus; CsA—cyclosporin A. 1. Antigen-presenting cell presents an antigen to a T cell, leading to an inflammatory response 2. Dephosphorylation of NFAT complex by calcineurin, a Ca^2+^ ion-dependent phosphatase. 3. Activation of IL-2 transcription through active NFAT complex. 4. IL-2 production 5. Induction of the CD25 receptor by IL-2, leading to the activation of the mTOR signaling pathway (whose core consists of two protein complexes—mTORC1 and mTORC2), which leads to T-lymphocyte proliferation and IL production. 6. CsA binds to cyclophilin to form a complex that inhibits calcineurin. 7. FK-506 binds to FK-BP, leading to the inhibition of calcineurin. 8. RAP inhibits the mTOR pathway. 9. AZA and MMF inhibit DNA synthesis, blocking proliferation and IL production. 10. Active GCR receptor complex inhibits COX-2 transcription. 11. Active GCR receptor complex stimulates lipocortin production, which leads to the inhibition of PLA_2_.

The mechanism of action of CsA is based on a specific and reversible inhibitory effect on T lymphocytes (Figure 1), which does not inhibit hematopoiesis or affect the body’s phagocytes. These reactions mainly involve the calcineurin pathway (nuclear factor of activated T cells), the activation of c-Jun N-terminal kinase (JNK), and p38 signaling pathways [3]. Cytosolic cyclophilin A (CypA), which is an immunophilin that regulates protein maturation in the cell, plays an important role in the calcineurin inhibition pathway. The CypA–CsA complex binds to and inhibits the activity of calcineurin protein phosphatase, which is essential in the activation of T lymphocytes [4]. CsA also affects the primary cellular response to the antigen, which in turn is suppressed by its action. The inhibition of T lymphocytes in the reactions they mediate results in the suppression of the entire response as well as of the humoral response, which is T-dependent [5]. Most likely, CsA also inhibits the reaction or formation of nascent T-lymphocyte memory cells themselves [3].

The use of CsA in treatment may be associated with a number of harmful side effects due to its high toxicity [3]. As an immunosuppressive drug, it has a number of serious side effects, including nephrotoxic, hepatotoxic, neurotoxic, cardiotoxic, hematologic, gastrointestinal, and cardiovascular effects [3,6]. It is likely that oxidative stress is one of the mechanisms of CsA toxicity. The actual evaluation of CsA toxicity is based on clinical monitoring and measuring its plasma concentration, but above all, evaluation requires the rigorous monitoring of renal and hepatic function and peripheral blood counts [5].

### 2.2. Tacrolimus

FK-506, like CsA, acts as a prodrug that becomes active only after binding to intracellular receptors or binding proteins: namely, immunophilins [7]. Its mechanism of action is mainly based on causing the inhibition of calcineurin (Figure 1), which is essential for the early activation of CD4+ helper T lymphocytes, by inhibiting at the transcriptional level the production of lymphokines (cytokines produced by T lymphocytes) necessary for cell growth and differentiation, mainly interleukin-2 [7,8]. The exact mechanism is much more complicated and begins when FK-506 binds to the drug-specific cytoplasmic immunophilin FKBP-12 (receptor protein), which is responsible for signal transduction in the cell. FK-506 binds to it and then forms a complex with Ca^2+^ contained in calmodulin and calcineurin, inhibiting the Ca^2+^-dependent signal transduction cascade in T lymphocytes. The resulting complex, by inhibiting the activity of calcineurin phosphatase, inhibits NF-AT (nuclear factor of activated T cells) and OAP (octamer-associated protein), which is an essential T-lymphocyte-specific transcription factor. This leads to IL-2 expression, which initiates T-cell activation signaling by entering into the nucleus and binding to a DNA complex that promotes the transcription of the IL-2 gene [7,8]. FK-506 prevents NF-ATc translocation into the nucleus, thereby preventing the transcription, synthesis, and secretion of interleukins 2, 3, 4, 5 and other cytokines such as GM-CSF, TNF-α and IFN-γ [7,8]. In addition, the inhibition of T lymphocyte activation by FK-506 results in the inhibition of Ca-dependent B lymphocyte proliferation [7].

The immunosuppressive mechanism of FK-506 is similar to that of CsA. Due to its metabolism and narrow therapeutic window, the administration of the drug is associated with frequent monitoring of the patient and dose adjustment. Like CsA, FK-506 exhibits high toxicity [8]. The main adverse effects of FK-506 are neurotoxicity, nephrotoxicity, hypertension, diabetogenic effects, and infectious complications [7].

### 2.3. Sirolimus/Rapamycin

RAP is an immunosuppressive and antiproliferative agent. Despite its similar structure to FK-506 and ability to bind to the FKBP124 receptor, its mechanism of action is different. Sirolimus, as an inhibitor, forms the RAP–FKBP12 complex, which does not affect calcineurin phosphatase but binds to effector TOR (target of rapamycin) proteins, which were first discovered in rats. In humans, the abbreviation mTOR (mammalian targets of rapamycin) is most often used [9].

mTOR consists of two distinct cell signaling complexes. mTOR complex one (mTORC1) is sensitive to RAP, while complex two (mTORC2) is resistant to it. mTORC1 consists of consecutive nodes of signaling pathways that phosphorylate various substrates responsible for cell growth, metabolism, autophagy, or proliferation. When RAP binds to the FKBP12 receptor to form the FKBP12–RAP complex, it binds to the FKBP12–RAP binding site of mTOR kinase, blocking the ability of substrates to bind to sites on mTORC1 that are responsible for the metabolic and proliferative pathways of the cell [10].

Cytokines, such as in the IL-2 and CD28/B7 costimulatory interaction pathway, activate mTOR, causing the dysregulation of the cell cycle by inhibiting DNA and protein synthesis. The immunosuppressive effect of RAP is due to the inhibition of leukocyte activity caused by T-cell proliferation being blocked, which is induced by cytokines such as IL-1/2/3/4/6/7/12/15. RAP has also a suppressive effect on NK cells and inhibits B-lymphocyte proliferation independent from Th lymphocyte activity. In addition, it has been noted that RAP does not affect IL-2-induced T lymphocyte apoptosis and indirectly reduces immunoglobulin synthesis by inhibiting cell differentiation into antibodies that are dependent on IL-2 and IL-6 cytokines [9,10].

The most common side effect of the drug is hyperlipidemia, which occurs in 60–80% of transplant patients treated with mTOR. This carries with it a 50-fold increase in the risk of cardiovascular events after kidney transplantation. The second most common complications are myeloid complications such as anemia, thrombocytopenia, and leukopenia. It has also been shown that an mTOR receptor inhibitor can significantly reduce fibrosis, which is a key component of successful wound healing [11,12].

### 2.4. Mycophenolate Mofetil

MMF is a prodrug, the morpholinoethyl ester of mycophenolic acid (MPA) isolated from *Penicillium* culture [13]. After administration, MMF is metabolized by almost complete hydrolysis into the active substance MPA, which binds to plasma albumin [14]. It has no effect on the synthesis of cytokines (IL-1 and IL-2). There are two pathways of action of the drug, both of which affect purine synthesis pathways.

The first is the de novo pathway. Its mechanism of action is based on a cytostatic effect on T and B lymphocytes (Figure 1), which it achieves by inhibiting the enzyme inosine monophosphate dehydrogenase (IMPDH), which is involved in the synthesis of guanosine nucleosides, essential DNA building blocks in lymphocyte proliferation [13]. MPA is a potent, selective, reversible, non-competitive inhibitor of type II IMPDH, specific to B and T lymphocytes [13,15]. As a result of the reduced production of PG, the transfer of saccharide molecules to glycoproteins that recruit monocytes and lymphocytes to sites of inflammation is slowed down, and the DNA synthesis and proliferation of T and B lymphocytes are inhibited. This results in a reduction in the number of lymphocytes and monocytes, without reducing the number of neutrophils, in the inflammatory focus [13]. In addition, MPA has been shown to increase apoptosis in activated T lymphocytes and inhibit the ability of dendritic cells to present antigens to T lymphocytes [15].

The second pathway for GMP production that is not synthesized in lymphocytes is the salvage pathway involving phosphoribosyl pyrophosphate (PRPP) and guanine, which are converted to GMP by hypoxanthine guanine phosphoribosyl transferase.

MMF is well tolerated by the body, and mild side effects are mainly limited to gastrointestinal abnormalities [13]. There are also data to show that MMF helps in the treatment of autoimmune diseases such as lupus erythematosus, myasthenia gravis, and glomerular disorders [16].

### 2.5. Azathioprine

AZA, 6-(1-methyl-4-nitroimidazole)-thiopurine, is an inactive purine. It is converted into the active metabolites mercaptopurine (6-MP) and thioguanine (6-TGN) when under the influence of enzymes such as hypoxanthine-guanine phosphoribosyl transferase (HPRT) and thiopurine methyltransferase (TPMT) [17].

Metabolized AZA is a slow-release prodrug of 6-mercaptopurine (6-MP). 6-MP is inactive but converts to various forms of active metabolites. It acts as a purine antagonist. It has become an immunosuppressive agent because by interfering with intracellular biochemical processes involving endogenous purines, it inhibits the production of antibodies as well as cell proliferation in general, including T lymphocytes. Endogenous purines are essential components for the construction of DNA, RNA, and coenzymes [18]. A metabolite such as 6-TGN is incorporated into replicating DNA or RNA, arresting cell proliferation [17]. Cytotoxic thioguanine nucleotides affect the restriction of purine nucleotides by inhibiting the amidotransferase enzyme pathway and swapping the configuration of the substituents (interconversion) of purine ribonucleotides [19].

AZA has different mechanisms of action (Figure 1). After conversion to 6-MP, it can be further catabolized or undergo intracellular anabolic processes, leading to the formation of thiopurine ribonucleosides or ribonucleosides [20]. AZA and the 6-mercaptopurine derivative mercapto–imidazole have also been shown to reduce T-cell proliferation and the nuclear factor signaling of activated T cells (NFAT). 6-MP alone affects T-cell migration by affecting cell adhesion, and AZA can directly initiate cell apoptosis and inhibit cell proliferation pathways [19].

Common side effects include nausea; fever; fatigue; joint pain; bone marrow suppression, including pancytopenia, thrombocytopenia and leukopenia (which are related to 6-TGN levels); rash; hepatotoxicity; infections; and kidney damage [17].

### 2.6. Glucocorticoids

GCSs belong to a class of steroid hormones called corticosteroids [21]. GCSs are widely used and are the most effective treatment for inflammatory and immune diseases. The effects of corticosteroids include: the inhibition of vasodilation occurring during ongoing inflammation; a reduction in vascular permeability, and thus, a reduction in leukocyte migration to the site of ongoing inflammation; and an effect on cell differentiation in the subsequent response of the body [22]. GCSs have two signaling pathways: the genomic and extragenomic pathways (Figure 1) [21].

In the case of the genomic pathway, once the receptor binds to the ligand, the protein undergoes conformation and reorganization to move through the nuclear pores into the nucleus. Once in the nucleus, it binds to the DNA-binding part and stimulates the expression of target genes [21]. GCSs can inhibit gene expression by neutralizing transcription factors from the cytoplasm responsible for the activation of genes that encode proteins used in the inflammatory process.

The extragenomic signaling pathway relies on there being a GCR in the cell membrane (mGCR) for GCSs, affecting equal signaling pathways. GCSs, as lipophilic compounds, easily build into the lipid cell membrane, causing changes in its physicochemical properties. They affect the regulation of calcium, sodium–potassium, and chloride channels. In addition, ion channels affect the fluidity of the cell membrane. The disturbance of its fluidity may cause, for example, the disruption of neutrophil degranulation [23].

The unusual impact of GCSs on inflammation, in contrast to non-steroidal anti-inflammatory drugs (NSAIDs), lies in their slightly different mechanism of action, in which, by inhibiting the release of arachidonic acid from membrane phospholipids, not only cyclooxygenase products are inhibited (as with NSAIDs), but also lipoxygenases [24]. By inhibiting phospholipases, GCSs prevent the biosynthesis of pro-inflammatory eicosanoids, including prostaglandins, leukotrienes, and hydroxy acids. As a result of GCSs binding to the receptor, translational and transcriptional changes occur, alongside the production of proteins with antiphospholipase activity [25]. GCSs inhibit the expression of genes that are responsible for creating or supporting inflammatory processes, such as pro-inflammatory cytokines, chemokines, and enzymes involved in initiating or sustaining the inflammatory response [26].

### 2.7. Basiliximab and Anti-Thymocyte Globulin

BAS is a chimeric monoclonal antibody used in current drug protocol to prevent acute graft rejection. It is an antibody directed against the CD25 molecule of the receptor for interleukin 2 (IL-2), which is found on activated T lymphocytes [27]. The drug has found use in heart transplantation as an induction of immunosuppression, preventing the acute rejection of the transplanted organ [2]. BAS has a range of side effects, including a greater predisposition to infection, anaphylactic reaction, nausea, constipation, and many others [28].

Polyclonal ATG is also used in current drug protocols as an induction of immunosuppression [2]. These globulins are isolated from rabbit serum. ATG targets T and B lymphocytes, as well as dendritic and NK cells. The protein binds to the target cell, inducing its apoptosis and impairing its ability to fuse with the vascular endothelium. The properties of ATG cause it to reduce the number of lymphocytes in the bloodstream, preventing the acute rejection of the transplanted organ [29]. The most common side effects of ATG administration are fever, chills, and lymphopenia [30]. However, more detailed analysis is needed of the cardiovascular effects of this drug.

## 3. Effects of Immunosuppressive Drugs on Various Components of the Cardiovascular System

### 3.1. Fibrosis and Myocardial Remodeling

Fibrosis and the remodeling of myocardial structure in heart transplant patients is a fairly common phenomenon, which can lead to shortened graft survival and the progressive loss of graft function [31]. It has been speculated that this phenomenon may be induced in patients who receive immunosuppressive therapy, as these drugs induce oxidative stress and may damage myocardial cells or contribute to myocardial fibrosis [32].

CsA has many proven cardiovascular side effects, alongside its impact on myocardial remodeling [33]. In rats receiving CsA at a dose of 15 mg/kg for 21 days, the myocardium underwent remodeling. Increased cardiac fibrosis; an increase in the expression of metalloproteinase 2, responsible for tissue remodeling; and an increase in the expression of vascular endothelial growth factor were observed. This phenomenon is presumably a response to the damaging effects of CsA [34]. Myocardial fibrosis and remodeling were also noted in another experiment. Comparisons were made between healthy rats, rats with a heart allograft, and transplanted rats receiving additional CsA. In this study, the drug reduced the inflammatory response in the heart, improving the histological structure of the graft, while leading to increased fibrosis [35]. However, most of the reports demonstrating the profibrogenic effects of CsA on myocardium involve either rats or cell cultures [36,37,38]; there are only a few older publications that have found fibrosis of the transplanted heart in patients taking CsA [39,40].

FK-506 is not a well-studied molecule in terms of its ability to cause fibrosis or myocardial remodeling. In minipigs with induced myocardial infarction, FK-506 reduced the infarct zone compared to the placebo group, reduced the loss of left ventricular function, and reduced fibrosis and cardiac remodeling after infarction. It had an inhibitory effect on the synthesis of α-smooth muscle actin, transforming growth factor-β, metalloproteinase-9, and tumor necrotic factor [41]. However, in cell cultures, FK-506 and RAP have been shown to reverse the inhibitory effect of the p38-MAPK pathway, resulting in increased collagen deposition in the heart and increased fibrosis [42]. More research on this topic is needed to accurately understand the effects of FK-506 on cardiac remodeling and its profibrotic properties.

There are few reports on the effects of RAP on cardiac remodeling and fibrosis, although one report indicates that the drug enhances the fibrosis of this organ in rats after nephrectomy. It has been noted that in the left ventricular myocardium, RAP (0.2 mg/kg/d) statistically significantly reduced the fibrotic zone in rats after nephrectomy, and the expression of type I collagen was lower. The hearts of rats that did not undergo nephrectomy were histologically almost no different from those of rats that additionally received RAP [43]. In other rats that received RAP for a period of 3 months, there were no changes in the amount of type I and III collagen in the myocardium, which would indicate a profibrogenic effect of the drug [44]. However, as with FK-506, more studies should be conducted on human myocardium.

The lack of reports on AZA does not allow for a conclusion to be drawn about the effect of this drug on the myocardium. However, a study was conducted in which the authors highlighted the effect of MMF as a drug that reduces the influx of lymphocytes and macrophages, decreases the synthesis of pro-inflammatory cytokines, and reduces fibrosis [45]. However, the drug requires further study to determine its effects on the myocardium.

There are a number of publications in which the authors have noted the effects of GCSs on the myocardium. In rats receiving dexamethasone at a dose of 350 µg/kg/d for 15 days, the weights of the hearts increased by 25% compared to the control group. In addition, left ventricular remodeling and increased fibrosis were observed in the hearts of rats receiving GCSs compared to the control group [46]. In another of the experiments, in dogs, prednisolone at a dose of 2 mg/kg/d for 84 days caused increased cardiac fibrosis, decreased levels of GCRs, and increased levels in the mineralocorticoid receptor compared to a control group [47]. High doses of GCRs may contribute to earlier cardiac aging, cardiac hypertrophy, and fibrosis, as well as progressive diastolic dysfunction, increased ventricular wall stiffness, and calcium imbalance in cardiomyocytes. [48,49]. In contrast, GCRs at physiological concentrations regulate normal cardiac development. It has been shown that the hearts of mice lacking the receptor for GCRs are unable to develop properly—their early development is no different from other mice, although they die prematurely [50]. As with the effects of GCSs on other aspects of the cardiovascular system, a relationship between the incidence of GCS-induced heart disease and the dose taken can be seen here as well.

### 3.2. Endothelial Function

The endothelium is a single layer of cells lining the blood and lymph vessels. Endothelial cell dysfunction is an important pathophysiological mechanism of many cardiovascular diseases. One of the key functions of the endothelium is the control of vascular tone, which it achieves by participating in the synthesis and release of mediators that relax blood vessels, such as prostaglandins or nitric oxide, and constrict them [51]. The disruption of this endothelial function leads to many diseases of the cardiovascular system, including stroke, myocardial infarction, chronic kidney disease, and others resulting from the impaired perfusion of the internal organs [52]. An equally important function of endothelial cells is their participation in the diapedesis of inflammatory cells to damaged tissues and the secretion of pro-inflammatory cytokines in response to the damaging factor, as well as their participation in the process of platelet aggregation following vascular injury.

Unfortunately, impaired endothelial integrity is a pathogenetic factor in atherosclerosis resulting from local inflammation, as well as from a generalized inflammatory response in some autoimmune diseases [53]. Therefore, it is necessary to look at commonly used immunosuppressive drugs and their effect on the vascular endothelium.

CsA can, as a side effect, cause damage to the vascular epithelium. When using CsA, it was found that endothelial cells release microparticles in response to damage that activate the alternative complement pathway, leading to the progression of vascular endothelial damage. A study on rats showed that complement activation by this mechanism may be one of the main causes of renal vascular damage and the resulting reduced renal function [54]. The use of CsA and FK-506 is also associated with the release of more pro-inflammatory cytokines by the vascular endothelium, which leads to cell damage and impaired endothelial function [55].

The toxicity of calcineurin inhibitors (CNIs) against the mouse vascular endothelium has been also proven. These drugs activate TLR4-dependent signaling, thus promoting the synthesis of pro-inflammatory cytokines [56]. In renal transplant patients receiving CNIs, the endothelial function of the brachial artery was evaluated. Reactive vasodilatation after the sublingual administration of nitroglycerin to these patients was significantly lower after CsA therapy, indicating damage to the vascular endothelium and dependent vasodilation. Endothelial damage also occurred in patients using FK-506, although to a lesser extent [57]. CNIs also modulate the vascular wall, leading to stiffening and fibrosis, which is most likely caused by increased oxidative stress and ongoing inflammation [32].

RAP does not directly damage endothelial cells, although it impairs its vasodilatory function. After exposure to acetylcholine, epithelial cells of the internal mammary artery treated with RAP showed a significant decrease in vasodilatory function, but microscopically, no differences were observed compared to the control group [58]. RAP also has an antiproliferative effect, which is why it is widely used in vascular surgery—stents coated with it prevent the restenosis of blood vessels [59].

AZA, like RAP, inhibits the proliferation of vascular endothelial cells. It has been shown to reduce endothelial cell proliferation in a dose-dependent manner and to increase the intracellular number of purine nucleotides, disrupting the balance of endothelial nucleotide [60]. One of the positive effects of AZA on blood vessels is a reduction in inflammation in the vessel walls and the inhibition of lymphocytes involved in the degradation of the integrity of the vessel walls. The use of AZA significantly reduced the incidence of aneurysms in mouse models of aorta [61].

A positive effect on the vascular endothelium can also be seen with the use of MMF. In combination therapy with CsA, this drug showed a preventive effect against vascular epithelial dysfunction caused by CsA toxicity compared to CsA monotherapy. This phenomenon is due to the increased availability of nitric oxide (NO), associated with the improved functionality of endothelial nitric oxide synthase [62]. Another study showed that MMF blocked the passage of immune cells through the walls of blood vessels. This leads to a reduction in the inflammatory infiltration in the transplanted organs, preventing their rejection. A decrease in the expression of E-selectin and P-selectin by endothelial cells during MMF treatment and interference with the synthesis of adhesive ligands by T lymphocytes have been shown [63].

GCSs also influence endothelial cell function. Animal studies have shown that, under physiological conditions, vascular vasodilatory function decreases after GCS administration, impairing the availability of NO as a result of the inhibition of NO synthase [64]. However, in human studies, there are no unequivocal reports demonstrating a negative effect of GCSs on endothelial function. A study on patients taking oral prednisolone for a month showed no impairment of endothelial function [65]. The positive effect of GCSs on endothelial function may be attributed to their mechanism of inhibiting the immune response in the inflammatory process. GCSs inhibit immune response pathways in the inflammatory process and enhance the synthesis of protective factors for the vascular epithelium. The integrity of vascular endothelial cells is also enhanced, and the ability to release phospholipids, which are substrates for the synthesis of inflammatory mediators, is reduced [66]. In contrast, one study showed that the use of GCSs in low doses for a year can improve endothelial function [67], although more research is needed to understand the effect of this class of drugs on the vascular endothelium.

### 3.3. Hypertension

Hypertension is one of the most important problems of modern times. It is the cause of many cardiovascular diseases—ischemic heart disease, stroke, chronic kidney disease, and others [68]. The incidence of hypertension in renal transplant patients is also common. This is a factor of both increased predisposition to cardiovascular disease and shortened allograft survival [69]. It is important to analyze whether hypertension is a result of the organ transplantation itself, or whether it may be caused by immunosuppressive therapy in transplant patients.

Taking CsA has a proven effect on the occurrence of hypertension. Almost all reports mention its deleterious effects on blood pressure [70]. Its mechanism of action is to impair endothelial function by reducing the synthesis of vasodilatory factors and promoting the secretion of vasoconstrictive factors. This contributes to vasoconstriction, leading to the induction of hypertension and the reduced perfusion of internal organs [71]. Taking CNIs also causes the activation of the renin–angiotensin–aldosterone system, increased oxidative stress, and increased plasma sodium levels, contributing to the development of hypertension. In addition, both CsA and FK-506 promote the synthesis of TGF-beta, which is associated with progressive renal fibrosis, resulting in impaired glomerular filtration [72]. CsA-induced high blood pressure is common in organ transplant patients, so it is important to monitor blood pressure and select the appropriate therapy to increase graft survival and improve patients’ quality of life [73].

FK-506 itself has also been studied for its effects on blood pressure. In mice receiving the drug, a significant increase in blood pressure was observed, and this effect was associated with the almost complete inhibition of endothelial function related to the vasoconstrictor effect [74]. It is also recognized that FK-506-induced hypertension may result from increased oxidative stress and the activation of the angiotensin-II-dependent vasoconstriction pathway [75]. In rats, a similar effect of CsA and RAP on the induction of hypertension was observed, but different pathophysiological mechanisms leading to its development were noted. While the use of CsA was more associated with increased oxidative stress and the activation of the sympathetic nervous system, RAP use was more associated with effects on lipid profile and blood morphological changes [76]. It was also shown that in kidney transplant patients, RAP caused hypertension and nephrotoxicity to a lesser extent than CNIs. On the other hand, it was more likely to cause adverse effects such as hematological disorders and slowed wound healing [77].

There are no reports on the effect of AZA that report an increase in blood pressure, although some authors point to AZA causing, as a side effect, the calcification of blood vessels [78], which may be the cause of, e.g., isolated hypertension in the elderly. This is undoubtedly one of the risk factors for cardiovascular complications [79].

MMF, on the other hand, reduces systolic and diastolic blood pressure. Patients with psoriasis and rheumatoid arthritis (RA) who took the drug regularly and suffered from stage I hypertension showed a significant decrease in blood pressure. The mechanism of action of MMF has been linked to a reduction in oxidative stress and a reduction in inflammatory cell infiltration in the kidneys [80]. Another study showed that MMF slightly reduced patients’ diastolic blood pressure and had almost no effect on systolic blood pressure [81]. Therefore, it can be concluded that this drug does not adversely affect blood pressure values, while it can reduce oxidative stress and preserve normal renal function, which may reduce the risk of hypertension in organ transplant patients and those suffering from autoimmune diseases.

Endogenous GCSs taken by humans at physiological concentrations regulate blood pressure [82]. In the case of GCS hypersecretion syndromes or in patients treated with these drugs for a long time, most people develop hypertension and other cardiovascular diseases [83]. In patients with hypertension, no significant differences were found in blood pressure measurements before and after taking GCSs. Short-term GCS therapy has been shown to have almost no effect on blood pressure [84]. Other authors showed a 17% increase in the risk of hypertension in patients whose daily dose exceeded 7.5 mg of GCS per day. Below this dose, no changes were observed [85]. This may be due to the chronic use of high doses of GCS. This claim is also supported by a study in which chronic oral GCS was administered to patients with various disease entities. Of the 71,642 patients, 24,896 developed hypertension over 6.6 years. Cumulative doses of GCS were associated with a significant increase in predisposition to the development of hypertension [86]. Chronic use of GCSs is associated with an increased predisposition to the development of hypertension, although the short-term use of GCSs does not significantly affect the development of hypertension.

### 3.4. Atherosclerosis

Atherosclerosis is an inflammatory disease of the arteries that is the main cause of cardiovascular disease [87]. The first stage of atherosclerotic plaque formation is the oxidation of LDL-C, leading to damage to the vessel wall, inducing inflammation. As a result, the inner wall of the arteries thickens; fatty plaques, monocytes, T lymphocytes, and platelets accumulate inside it; and smooth muscle proliferation occurs [88]. The rupture of the formed atherosclerotic plaque leads to the partial or complete thrombotic occlusion of the vessel, resulting in ischemia of the organ in question and necrosis. It is the most important factor in the pathogenesis of ischemic heart disease [89]. Analyzing the effect of immunosuppressive drugs on lipid profile and endothelial function as well as the predisposition to oxidative stress, which are the basis for the formation of atherosclerotic plaques, one can presume their influence on atherogenesis.

CsA increases oxidative stress, contributing to oxygen free-radical-related vascular damage [90]. Thus, the effects of CsA on blood vessels, combined with its hyperlipemic effects, can be considered to promote an extremely high risk of atherosclerosis. FK-506, on the other hand, does not significantly impact the development of dyslipidemia, but has a similar profile of action on the oxidative status of the body to CsA. After kidney transplantation, patients who received FK-506 or CsA had a significantly increased oxidative status compared to a group of healthy people, although no differences were found between people receiving FK-506 or CsA [91].

In FK-506-treated mice, a reduction in atherosclerotic plaques, the thinning of the inner walls of the vessels, and the absence of clear cholesterol crystals were observed [92]. Therefore, due to the limited number of studies, it is difficult to draw conclusions about the atherogenic effects of this drug in humans.

RAP, on the other hand, has been recognized as a drug that prevents vascular atherosclerosis. In animal models, RAP stabilized the atherosclerotic plaque and reduced the necrotic core of the plaque and vascular inflammation [93]. Similarly, in old mice with advanced atherosclerotic lesions receiving RAP, it was noted that the drug contributed to an increase in vascular blood flow that was comparable to young individuals [94].

It has also been proven that AZA has anti-proliferative and anti-atherosclerotic effects. Its metabolite 6-mercaptopurine inhibits the expression of monocyte chemoattractants, reducing their influx into the forming atherosclerotic plaque, thereby reducing inflammation in the plaque. In mice, this feature reduced atherosclerotic lesion formation [95].

MMF, on the other hand, has an ambivalent effect. A significant reduction in diet-induced atherosclerotic potential was observed in rabbits receiving MMF. These changes were justified by a reduction in macrophage infiltration and a reduction in smooth muscle cell proliferation in atherosclerotic plaques [96]. Similar results were obtained in patients with symptomatic carotid artery stenosis. After 2.5 weeks of administration of MMF at a dose of 1000 µg, reduced infiltration of inflammatory cells into atherosclerotic plaques and reduced expression of pro-inflammatory genes were observed in patients [97]. In contrast, lupus patients displayed no changes in atherosclerosis with chronic administration of MMF [98]. Thus, this drug requires further research in terms of its effect on reducing atherogenesis.

GCSs regulate lipid metabolism in the body at physiological concentrations. In diseases associated with inadequate GCS secretion, there is a greater predisposition to lipid disorders, the development of atherosclerosis, and cardiovascular events [99]. In patients with autoimmune diseases taking GCSs, the predisposition to atherosclerosis is higher, but it is not known whether this is due to the ongoing inflammatory process or to drug intake. In animal models, prednisone/prednisolone administration slowed the progression of atherosclerosis, a process that has been substantiated by the inhibitory effect of GCSs on monocyte recruitment in the atherosclerotic plaque. In mouse models, however, the opposite phenomenon was demonstrated—these animals, after stimulation with stress stimuli, secreted higher doses of corticosterone, which was the reason for the more frequent occurrence of atherosclerotic lesions. Clare MacLeod et al. have argued that GCSs cannot be perceived as a factor predisposing or limiting the process of atherogenesis, but steroid-regulated cellular mechanisms should be taken into account [100]. Therefore, more research should be conducted to find out how GCSs affect increased cardiovascular risk and to consider the benefits and disadvantages of taking these drugs in a time- and dose-dependent manner [101].

### 3.5. Dyslipidemia

Dyslipidemia is a disorder of the body’s lipid homeostasis, manifested primarily through increased levels of total cholesterol (TC), low-density lipoprotein cholesterol (LDL-C), triglycerides (TG), and decreased levels of high-density lipoprotein cholesterol (HDL-C) [57]. This disease is one of the main causes of coronary heart disease and strokes. The protective effect of a normal lipid profile in the prevention of cardiovascular disease has been proven [102]. It is commonly believed that internal organ transplantation is a significant risk factor for dyslipidemia, which is one of the components of the metabolic syndrome [103].

Immunosuppressive drugs may contribute to an increase in the incidence of this disease entity in patients using them [104]. Among patients who had undergone kidney transplantation, significant increases in TC, LDL-C, and TG were observed in a CsA-treated group, while no significant changes in the lipid profile were observed in patients taking FK-506. This phenomenon was correlated with the effect of CsA on CD36 expression on peripheral blood monocytes [105]. In a mouse study, CsA was shown to be involved in abnormal lipoprotein clearance, decreased lipoprotein lipase activity, elevated apolipoprotein C-III, and impaired subtilisin/kexin type 9 protein-converting activity, thus negating the negative impact of CsA on the expression of low-density lipoprotein receptors (LDLrs) [106]. It is believed that CsA significantly affects the lipid profile, while FK-506 does not contribute to hypercholesterolaemia [107], although there are reports that taking high doses of FK-506 is a factor in the development of dyslipidemia in liver transplant recipients [108].

RAP has been identified as a key factor in disrupting the lipid profile in renal transplant patients. Similar increases in TG and TC were observed in renal transplant patients taking RAP alone or RAP plus FK-506 [109]. Another study of kidney transplant recipients showed an increase in TC of over 50% and in TG of approximately 95% in patients who received RAP as part of immunosuppressive therapy [110].

In contrast, AZA and MMF do not affect lipid metabolism. In patients taking CsA with GCSs and AZA or MMF in the first year after kidney transplantation, both groups showed similar increases in TC and TG, regardless of whether their therapy included AZA or MMF. However, this increase was associated with the hyperlipemic effects of CsA and GCSs [111]. There are also no significant reports in the literature on the negative effect of AZA alone on the lipid profile [112], and MMF intake is not a significant risk factor for the development of dyslipidemia [113].

GCSs, on the other hand, have a proven negative effect on the lipid profile. In patients with Cushing’s syndrome, increases in TG and TC are observed, which can be explained by the direct effect of steroids on lipolysis, free fatty acid turnover, very-low-density lipoprotein (VLDL) synthesis, and fat accumulation in the liver [114]. Renal transplant patients treated with high doses of GCS developed hypertriglyceridemia, while reducing the dose of GCS improved the lipid profile [115], which may suggest that these drugs have a dose-dependent effect on lipid metabolism. Healthy men were administered prednisone at a dose of 0.35 mg/kg/d for two weeks. After this time, significant increases in VLDL-TG and VLDL-C with a concomitant increase in HDL-C were observed. All values returned to normal after GCS discontinuation [116].

### 3.6. Hyperglycemia

Type II diabetes, which is one of the most common chronic diseases in the world, has become a serious problem for both patients and physicians [117]. Cardiovascular incidents, such as myocardial infarction and stroke, are the most common cause of death in patients with this disease [118]. One of the important complications of diabetes is diabetic nephropathy, which leads to chronic kidney disease along with reduced glomerular filtration fraction resulting from damage to small vessels [119]. Renal damage and the occurrence of cardiovascular complications significantly affect the survival rate of patients after organ transplantation. Therefore, the impact of immunosuppressive drugs taken in the post-transplant period on carbohydrate metabolism in the human body should be examined.

Calcineurin inhibitors were analyzed for their effect on the likelihood of hyperglycemia and pre-diabetic conditions. FK-506 is more likely to cause impaired glucose tolerance in kidney transplant patients than CsA—18% versus 8%. Taking FK-506 is also associated with a 33% increase in the likelihood of being in a pre-diabetic state 12 months after kidney transplantation. The negative effect of these drugs on glucose metabolism has also been demonstrated in animal models [120]. A similar phenomenon was observed in children taking CsA or FK-506—diabetes developed significantly in those taking FK-506 [121].

RAP, when administered to transplant patients, has a hyperglycemic effect, but this relationship has been linked to the combination of this drug with other immunosuppressive drugs, such as calcineurin inhibitors. In oncology patients, the administration of RAP is associated with mild, reversible hyperglycemia, while in healthy individuals, no significant increase in blood glucose concentration has been observed [122].

There are few reports on the hyperglycemic effects of AZA and MMF. Studies have not shown increased blood glucose levels in patients taking these drugs [123,124].

GCSs taken by patients cause insulin resistance, increased gluconeogenesis, and decreased insulin secretion [121]. However, for diabetic and non-diabetic patients, taking GCS leads to hyperglycemia. The risk of carbohydrate metabolism disorders in patients taking GCSs depends on the dose of the drug taken and the duration of therapy [125]. In hematology patients taking high doses of GCS, diabetes developed in 40.6% of them within 2–3 months; however, in most patients, it stabilized spontaneously after some time. This shows the high potential of GCSs in inducing hyperglycemia [126]. Despite the incidence of hyperglycemia after steroid therapy, it is not fully known how this condition affects comorbidities and mortality. However, caution should be exercised when administering GCSs for chronic treatment, and greater emphasis should be placed on methods of diabetes prevention through diet and physical activity [127].

### 3.7. Metabolic Syndrome

Metabolic syndrome (MS) describes a group of metabolic diseases that increase the risk of cardiovascular disease. It includes abdominal obesity, hypertension, and carbohydrate and lipid metabolism disorders. The pathogenesis of MS may result from increased oxidative stress and a systemic inflammatory response to harmful factors [128]. Immunosuppressive drugs affect many aspects of health, leading to hypertension, endothelial dysfunction, and carbohydrate and lipid metabolism disorders, so they may also contribute to the development of MS.

CsA, like FK-506, increases the risk of metabolic disorders related to lipid metabolism. CsA therapy may lead to increases in TG, TC, and LDL-C levels, while increasing the likelihood of abdominal obesity and cardiovascular disease. FK-506 also leads to lipid disorders, though to a lesser extent than CsA [129,130]. Similarly, these drugs affect carbohydrate metabolism. It has been shown that taking CNIs is associated with an increased risk of hyperglycemia and diabetes [120,121]. Increased oxidative stress and the adverse effects of CNIs on blood vessels also contribute to the increased incidence of hypertension, one of the components of MS [70,71,72]. Unfortunately, there are no conclusive studies on the effects of CNIs on weight gain in humans. Thus, CNI drugs may contribute significantly to the increased risk of developing MS and the increased incidence of cardiovascular disease.

RAP also has a negative effect on lipid metabolism, increasing the secretion of TG and VLDL in the liver. The drug has time- and dose-dependent adverse effects on lipid metabolism [110]. However, RAP does not have a significant effect on carbohydrate metabolism, the disorders of which are an important component of MS [122]. The effect of RAP on the induction of hypertension is also unclear. This makes it impossible to draw clear conclusions about this component of MS [77,131]. In contrast, another study showed that chronic RAP intake has a beneficial effect on lipid metabolism, reduces insulin resistance, and leads to weight loss. However, this study was conducted on a mouse model and not on humans [132]. The impact of RAP on the risk of developing MS remains unclear and requires further research in this area.

AZA and MMF have no effect on lipid and carbohydrate metabolism, while they reduce the risk of MS [111,112,113,123,124]. AZA also has no significant effect on blood pressure, while MMF reduces oxidative stress and preserves glomerular function, thus reducing the risk of hypertension [78,79,80,81]. Neither AZA nor MMF increase the risk of MS in patients taking these drugs.

GCSs negatively affect lipid and carbohydrate metabolism. They increase the levels of TG, TC, VLDL, and glucose in the blood. However, this effect is dose-dependent and can be reduced by decreasing the doses of GCS taken [114,115,116,121,125,126,127]. Long-term use of GCSs may also lead to the development of hypertension, which is an important cardiovascular risk factor and is also part of MS [83,85,86]. These drugs are also responsible for the development of obesity in patients using them [133]. The negative impact of GCSs on carbohydrate and lipid metabolism, blood pressure, and body weight leads to the development of MS, which adversely affects the cardiovascular system [134,135]. Unfortunately, GCSs are drugs that are widely used in transplantology and are one component of induction and maintenance therapy, so their chronic use with other drugs, such as CNIs, RAP, MMF, or AZA, will lead to the development of metabolic disorders and an increased risk of cardiovascular diseases.

### 3.8. Hyperuricemia

Hyperuricemia is a clinical condition in which urea concentration exceeds 6.0 mg/dL in women and 7 mg/dL in men. It is one of the factors that contributes to kidney and cardiovascular diseases. High blood urates are pathogenic in two ways: intracellularly, inducing oxidative stress, inflammation, smooth muscle cell proliferation, endothelial dysfunction (including impaired nitric oxide production) and the activation of the renin–angiotensin system; and extracellularly, leading to atherosclerosis and vascular calcification. In atherosclerosis, the presence of extracellular urate in the atherosclerotic plaque facilitates its formation, while intravascular urate deposition enables calcium aggregation [136].

In patients who had undergone allograft kidney transplantation, CsA intake was associated with an increased risk of developing hyperuricemia due to the drug causing vasoconstriction, increased endothelin-1 release, and decreased nitric oxide production by the vascular endothelium [137]. Similar results were obtained in patients who had undergone liver transplantation. During CsA treatment, hyperuricemia developed in 8 of 11 patients (72%), and after the administration of FK-506 in 9 of 48 (18%) patients [138]. In another study, the authors reported an increased incidence and presence of multiple risk factors for the development of hyperuricemia following the use of CNIs after organ transplantation. They noticed a lower incidence of hyperuricemia after the administration of FK-506 than after CsA, despite a similar mechanism of action for the two drugs [139].

Determining the incidence of hyperuricemia after RAP administration is difficult to estimate because many treatment regimens are based on the combination of RAP and CsA. In renal transplant patients using a regimen without CsA, a reduced incidence of hyperuricemia was noted, indicating that RAP has no effect on uric acid metabolism [140]. However, due to the lack of publications determining the effect of RAP on blood urate concentration, no clear conclusions can be drawn.

AZA may reduce the concentration of urate in the blood. In patients suffering from chronic gout, AZA displayed a good effect in combination with pegloticase, leading to a decrease in blood urate concentration [141]. The available research on the effect of AZA on urate concentration may be outdated because it was conducted in the 1960s, so more research should be conducted in this direction [142]. On the other hand, it should be remembered that AZA interacts with allopurinol, causing bone marrow suppression, and therefore, it should be used with caution in patients with hyperuricemia [139,143].

MMF, on the other hand, does not increase the level of urates in the blood or it leads to their reduction. In patients with renal dysfunction, in whom CNI therapy was replaced with MMF monotherapy, a reduction in the mean uric acid concentration was observed, while in the control group, the concentration remained above the norm [144]. It is not known whether this phenomenon is related to the harmful effect of CNIs on blood urate concentrations or to the protective effect of MMF. More studies need to be conducted in this area.

GCSs are used in most immunosuppressive treatment protocols, but there are no studies on the incidence of hyperuricemia when used as monotherapy. In one older study, patients were given prednisolone, which caused significant increases in blood urate levels. This phenomenon was explained by the catabolic effects of GCSs on proteins and their effect on nitrogen metabolism [145]. In turn, GCSs are drugs that reduce inflammation and can be used during an acute attack of gout [146]. However, there is concern that when the dose is reduced, the condition may worsen, meaning that a maintenance dose will be necessary [147].

### 3.9. Biological Drugs—Effects on Selected Metabolic and Cardiovascular Aspects

BAS used in heart transplantation affects the thickness of coronary vessels. Heart transplant patients who received BAS had reduced coronary vessel volume as well as a thinner intima layer compared to control patients [148]. In turn, a study on pregnant rats showed that BAS did not cause blood pressure to rise [149].

The negative effect of BAS on carbohydrate metabolism has also been demonstrated. Patients treated with BAS showed a statistically significant increase in the incidence of diabetes mellitus after organ transplantation compared to the group that did not take the drug, which may indicate that the drug disrupts carbohydrate metabolism [150]. Another study also confirmed the impact of BAS on the risk of developing diabetes after organ transplantation by comparing the drug with ATG. Patients have been shown to have a much greater predisposition to developing diabetes while taking BAS [151].

In acute cardiac ischemia-induced rats receiving ATG, a less extensive ischemic zone with fewer necrotic cells was observed compared to the control group. After 6 weeks of observation, the hearts of these animals showed much less remodeling and better blood supply. In this case, ATG enhanced the release of pro-angiogenic molecules and reduced ischemia-induced cell damage [152].

In another experiment on 43 patients treated with ATG, as many as 20 patients experienced post-treatment complications, of which 12 patients had hypotension. There were no therapy complications in the group taking BAS. It has been proven that BAS is as effective in causing immunosuppression as ATG, but has fewer side effects and is safer to use [153].

A study on mice showed that CD8+ T lymphocytes promote the development of atherosclerotic plaque through lipid accumulation, macrophages, and the apoptosis of smooth muscle cells and the vascular endothelium. Reducing the percentage of CD8+ T lymphocytes contributed to a decrease in lipid accumulation in the aortic wall [154]. In a retrospective study on kidney transplant patients, ATG was associated with an increased incidence of atherosclerosis. It has been noted that ATG contributes to a decrease in the percentage of CD4+ T cells, as well as an increase in the number of CD8+ T cells, which is associated with cardiovascular diseases and especially with a higher risk of atherosclerosis [155]. However, another study on heart transplant patients indicates a protective effect of ATG on the formation of atherosclerotic plaques [156].

## 4. Summary

Immunosuppressive drugs constitute a very diverse group of drugs. Immunosuppressive drugs should be selected carefully for people with multiple diseases so as not to worsen the clinical conditions of patients after organ transplantation. The adverse effects of immunosuppressive drugs on the cardiovascular system should be taken into account (Table 2).

## 5. Conclusions

The adverse effects of immunosuppressive drugs are associated with toxicity within the cardiovascular system, which may be a problem in the clinical management of patients after transplantation. Immunosuppressants act on the cardiovascular system in a variety of ways, including fibrosis and myocardial remodeling, endothelium disfunction, hypertension, atherosclerosis, dyslipidemia or hyperglycaemia, metabolic syndrome, and hyperuricemia. The use of multidrug protocols makes it possible to develop regimens that can reduce the incidence of cardiovascular events. A better understanding of their mechanisms of action and the range of complications could enable physicians to select the appropriate therapy for a given patient, as well as to reduce complications and prolong life.

## 6. Clinical Implications

**Figure 2:**
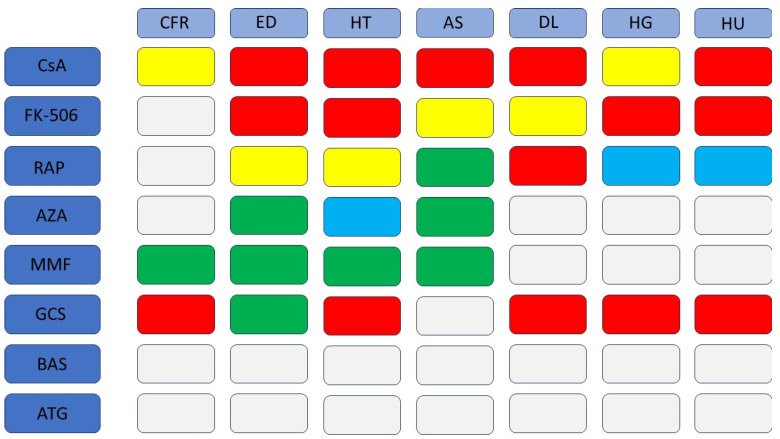
Effects of immunosuppressive drugs on the cardiovascular system—a clinical aspect. CFR—cardiac fibrosis and remodeling; ED—endothelial dysfunction; HT—hypertension; AS—atherosclerosis; DL—dyslipidemia; HG—hyperglycemia; HU—hyperuricemia; RED—the drug induces a given dysfunction—great caution should be exercised in patients with the particular disease; YELLOW—the drug may induce a given dysfunction—caution should be exercised in patients with the particular disease; GREEN—the drug has a positive effect on a given dysfunction; BLUE—no effect; WHITE—more research needed.

CNIs are drugs whose use is associated with numerous complications (Figure 2). CsA causes increased myocardial fibrosis, although this is primarily proven in animal studies. Reports of its profibrotic effects on the human heart date back to work before 2000, so this drug should be further investigated. FK-506 is similar in this regard—the few studies on its negative and positive effects do not allow clear conclusions to be drawn. A larger number of studies concern the effects of these drugs on the vascular endothelium. Both show negative effects on its function and can lead to endothelial cell damage. This is associated with a greater predisposition to the development of atherosclerosis (especially with CsA) in patients using CNIs, as well as hypertension, as shown in numerous review studies and human and animal experiments. Negative effects on lipid and carbohydrate metabolism are also attributed to CNIs, although studies show that with FK-506, the hyperglycemic effect is much greater than with CsA. In contrast, CsA shows a greater tendency to disrupt the lipid profiles of patients. In human studies, CsA and FK-506 have also been shown to increase serum uric acid concentrations and may contribute to gout. The possibility of developing metabolic syndrome should be particularly considered when using CNIs.

RAP is a drug that has fewer cardiovascular side effects than CNIs (Figure 2). It is not known whether it contributes to increased fibrosis or myocardial remodeling, so more research is needed to determine this relationship. However, it may contribute to a decrease in vascular diastolic function, resulting in the development of hypertension. Despite its adverse effects on vascular endothelial function, RAP stabilizes atherosclerotic plaque and may reduce organ ischaemia. In human studies, it has been shown to have negative effects on lipid metabolism, although it does not impair carbohydrate metabolism or affect uric acid metabolism.

Antimetabolites such as AZA and MMF are not well-known drugs in terms of cardiovascular complications (Figure 2). MMF, in one human study, reduced the synthesis of pro-inflammatory factors, reducing cardiac fibrosis. Both MMF and AZA increase NO-dependent vasodilation, with MMF reducing systolic and diastolic blood pressure. Antimetabolites stabilize atherosclerotic plaque and reduce vascular inflammation. Unfortunately, there are no studies that can conclusively determine the effect of these drugs on lipid and carbohydrate metabolism, or their effect on uric acid metabolism.

GCSs are a group of drugs that are commonly used not only in transplantation, but also in other areas of medicine. However, these drugs have a whole range of side effects that affect the cardiovascular system (Figure 2). Their use has been shown to be associated with pathological cardiac remodeling and fibrosis, leading to progressive loss of organ function. Despite their positive effects on the vascular endothelium, their chronic use and high doses lead to the development of hypertension. However, it is not known how GCSs affect the development of atherosclerosis. On the other hand, many experiments show that GCSs disrupt lipid and carbohydrate metabolism, as well as uric acid metabolism. The effect of these disturbances depends primarily on the duration of use and the dose of the drug. GCSs are also accompanied by an increased predisposition to the development of metabolic syndrome.

Single reports confirm the impact of BAS on the risk of developing diabetes. However, based on the available data, no clear conclusions can be drawn regarding the impacts of BAS and ATG on the cardiovascular system.

The optimal regimen of immunosuppressive therapy must be tailored to the individual patient and be selective and specific. The immunosuppressive drugs currently in use meet these assumptions. Calcineurin inhibitors primarily affect T lymphocytes, but they are associated with a number of side effects. In turn, mTOR kinase inhibitors inhibit the response to selected cytokines, which is associated with a decrease in selectivity. The use of drugs from other groups taken alongside CNIs in multi-drug protocols is often associated with a reduction in the negative effects associated with the use of CNIs; however, special attention should be paid to the dosage so that side effects are not exacerbated. It should be emphasized that caution should be exercised with regard to heart transplant patients who receive multiple immunosuppressive drugs, which are associated with a higher incidence of cardiovascular events. Therefore, in clinical practice, therapy should be optimized to take into account risk factors related to the functioning of the cardiovascular system (Figure 2).

## 7. Recommendations for Future Research

Despite the many studies conducted on human models, there is still a lack of information on the effects of some immunosuppressive drugs in the context of selected cardiovascular dysfunctions. Unfortunately, there are no studies on the effect of AZA or MMF on the metabolism of lipids and carbohydrates, or their effect on uric acid metabolism. It is also unknown how GCSs influence the development of atherosclerosis. Moreover, there is a clear need for research to determine the impact of BAS and ATG on selected parameters related to the functioning of the human cardiovascular system.

Large-scale pharmacoepidemiological studies on vascular organ transplant patients taking immunosuppressive drugs under different treatment strategies (e.g., monotherapy/multi-drug therapy/conversion from multi-drug therapy to monotherapy) to determine the risk for specific cardiovascular events would be helpful. Various criteria for dividing patients (e.g., age, comorbidities) should also be taken into account.

It is worth mentioning that regarding patients undergoing immunosuppressive therapy, it may be interesting to assess the role of adaptive immune cells, which are being highlighted in the development of cardiovascular disease. The available literature emphasizes the clinical significance of selected biomarkers and their predictive value. Selected biomarkers representing fibrosis, inflammatory processes, and vascular endothelial dysfunction also seem to be promising [157,158,159,160,161].

## Figures and Tables

**Table 1:** Immunosuppressants currently used in heart transplantation [2].

	Induction Therapy	Maintenance Therapy
Calcineurin Inhibitors	FK-506	CsA FK-506
mTOR Inhibitors	–	RAP Everolimus
Anti-metabolites	MMF	MMF Enteric-coated MMF Mycophenolate sodium AZA
Glucocorticoids	High-dose GCSs	Methylprednisolone Prednisolone
Biologic Agents	ATG BAS	–

ATG—anti-thymocyte globulin; AZA—azathioprine; BAS—basiliximab; CsA—cyclosporin A; FK-506—tacrolimus, GCSs—glucocorticoids; MMF—mycophenolate mofetil; RAP—sirolimus/rapamycin.

**Table 2:** The effects of immunosuppressive drugs on the cardiovascular system.

Disease Entity	Drug	Effect	Model	Reference
Fibrosis and Myocardial Remodeling	CsA	Increased cardiac fibrosis, increased expression of metalloproteinase 2, increased expression of vascular endothelial growth factor.	Rat	[34]
		Reduced inflammatory response, increased fibrosis.	Rat	[35]
		Profibrogenic effect.	Rat; CC *	[36,37,38]
		Profibrogenic effect.	Human	[39,40]
	FK-506	Reduced infarct zone, reduced loss of left ventricular function, reduced fibrosis, and cardiac remodeling after infarction.	Pig	[41]
		Increased collagen deposition in the heart and increased fibrosis.	CC	[42]
	RAP	Increased collagen deposition in the heart and increased fibrosis.	CC	[42]
		Reduced fibrotic zone in rats after nephrectomy, reduced expression of type I collagen.	Rat	[43]
		No changes.	Rat	[44]
	AZA	No reports.	---	---
	MMF	Decreased synthesis of pro-inflammatory cytokines and reduced fibrosis.	Human	[45]
	GCS	Increased weight of heart, left ventricular remodeling, increased fibrosis.	Rat	[46]
		Increased cardiac fibrosis, decreased levels of GCRs, and increased levels for the mineralocorticoid receptor.	Dog	[47]
		Earlier cardiac aging, hypertrophy, fibrosis, diastolic dysfunction, increased wall stiffness, calcium imbalance in cardiomyocytes.	R/M **	[48,49]
	ATG	Reduced ischemia-induced cell damage, reduced remodeling and necrosis.	Rat	[152]
Endothelial Disfunction	CsA	Activation of complement pathway, leading to endothelial damage.	Rat	[54]
		Release of proinflammatory cytokines, causing damage and impaired endothelial function.	CC	[55,56]
		Reduced vasodilatation.	Human	[57]
		Modulation of vascular wall, increased oxidative stress and inflammation.	R/M	[32]
	FK-506	Release of proinflammatory cytokines, causing damage and impaired endothelial function.	CC	[55,56]
		Reduced vasodilatation, to a lesser extent than CsA.	Human	[57]
		Modulation of vascular wall, increased oxidative stress and inflammation.	R/M	[32]
	RAP	Reduced vasodilatation.	Human	[58]
		Antiproliferative effect.	Human	[59]
	AZA	Antiproliferative effect.	CC	[60]
		Reduction in inflammation in the vessel walls and the inhibition of lymphocytes involved in the degradation of the integrity of the vessel walls.	Mouse	[61]
	MMF	Increased availability of NO associated with improved functionality of endothelial nitric oxide synthase.	Rat	[62]
		Reduction in inflammatory infiltration in the transplanted organs.	CC	[63]
	GCS	Reduced vasodilatation.	R/M	[64]
		No effect.	Human	[65]
		Inhibition of immune response pathways in the inflammatory process and enhancement of the synthesis of protective factors for the vascular epithelium.	R/M	[66]
		Improvement of endothelial function (low doses).	Human	[67]
	BAS	Reduced coronary vessel volume and thinner intima layer.	Human	[148]
Hypertension	CsA	Alteration of blood pressure.	R/M	[70]
		Increased vasoconstriction.	R/M	[71]
		Activation of renin–angiotensin–aldosterone system, increased oxidative stress, increased plasma sodium levels.	R/M	[72]
	FK-506	Activation of renin–angiotensin–aldosterone system, increased oxidative stress, increased plasma sodium levels.	R/M	[72]
		Inhibition of endothelial function related to the vasoconstrictor effect.	RM	[74]
		Increased oxidative stress and activation of the angiotensin-II-dependent vasoconstriction pathway.	Mouse	[75]
	RAP	Induction of hypertension.	Rat	[76]
		Induction of hypertension and nephrotoxicity to a lesser extent than CNIs.	R/M	[77]
	AZA	Calcification of blood vessels, causing isolated hypertension in the elderly.	CC	[78]
	MMF	Reduction in systolic and diastolic blood pressure.	Human	[80]
		Reduction in diastolic blood pressure, though almost no effect on systolic blood pressure.	R/M	[81]
	GCS	Development hypertension and other cardiovascular diseases.	R/M	[83]
		No effect on blood pressure (short-term use).	Human	[84]
		Increased blood pressure (prednisolone > 7.5 mg/d), no changes in blood pressure (<7.5 mg/d).	Human	[85]
		Significant increase in predisposition to the development of hypertension (cumulative doses of GCS).	Human	[86]
	BAS	No effect.	Rat	[149]
	ATG	Risk of hypotension.	Human	[153]
Atherosclerosis	CsA	Increased oxidative stress, contributing to oxygen free-radical-related vascular damage.	R/M	[90]
		Extremely high risk of atherosclerosis.	Human	[91]
	FK-506	Similar profile of action on the oxidative status of the body to CsA, not a significant cause of dyslipidemia.	Human	[91]
		Inhibition of atherosclerotic plaque development.	Mouse	[92]
	RAP	Stabilization of atherosclerotic plaque, reduced necrotic core of the plaque, and reduced vascular inflammation.	R/M	[93]
		Increased vascular blood flow in atherosclerotic vessels.	Mouse	[94]
	AZA	Reduced inflammation in the plaque, reduced atherosclerotic lesion formation.	Mouse	[95]
	MMF	Reduction in diet-induced atherosclerotic potential.	Rabbit	[96]
		Reduced inflammatory cell infiltration into the plaques and reduced expression of pro-inflammatory genes.	Human	[97]
		No decrease in carotid artery thickness (measure of atherosclerosis progression).	Human	[98]
	GCS	Unknown, but more research should be conducted.	R/M	[100,101]
	ATG	Increased risk of atherosclerosis.	Human	[155]
		Reduced progression of atherosclerosis.	Human	[156]
Dyslipidemia	CsA	Significant increase in TC, LDL-C, and TG.	R/M	[104]
		Abnormal lipoprotein clearance, decreased lipoprotein lipase activity, elevated apolipoprotein C-III, and impaired subtilisin/kexin type 9 protein converting activity.	Mouse	[106]
		Increased risk of hypercholesterolaemia.	R/M	[107]
	FK-506	No significant changes in lipid profile.	Human	[105]
		No contribution to hypercholesterolemia.	R/M	[107]
		Increased risk of dyslipidemia in patients taking high doses of FK-506.	Human	[108]
		Increased levels of TG and TC.	Human	[109]
	RAP	Increased levels of TG and TC.	Human	[109,110]
	AZA	No significant reports.	Human	[111]
		No significant reports	R/M	[112]
	MMF	No significant reports.	Human	[111]
		No significant reports	R/M	[113]
	GCS	Increased levels of TG an TC.	R/M	[114]
		Increased risk of hypertriglyceridemia (high dosages).	Human	[115]
		Significant increase in VLDL-TG and VLDL-C with a concomitant increase in HDL-C.	Human	[116]
Hyperglycemia	CsA	Impaired glucose tolerance.	R/M	[120]
		Increased risk of diabetes mellitus.	Human	[121]
	FK-506	Impaired glucose tolerance (more than CsA).	R/M	[120]
		Increased risk of diabetes mellitus (higher risk than CsA).	Human	[121]
	RAP	Mild hyperglycemia in oncology patients, no increase in blood glucose in healthy patients.	R/M	[122]
	AZA	No significant reports.	R/M	[123,124]
	MMF	No significant reports.	R/M	[123,124]
	GCS	Dose-dependent risk of carbohydrate metabolism disorders.	R/M	[125]
		High potential to induce hyperglycemia.	Human	[126]
	BAS	Greater predisposition to developing diabetes.	Human	[150,151]
Metabolic Syndrome	CsA	Large contribution to development of MS.	N/A ***	[70,71,72,120,121,129,130]
	FK-506	Large contribution to development of MS.	N/A	[70,71,72,120,121,129,130]
	RAP	More studies needed.	N/A	[77,110,122,131,132]
	AZA	No increase in risk of MS development.	N/A	[78,79,80,81,111,112,113,123,124]
	MMF	No increase in risk of MS development.	N/A	[78,79,80,81,111,112,113,123,124]
	GCS	Dose- and time-depended risk of MS development.	N/A	[83,85,86,114,115,116,121,125,126,127,133,134,135]
Hyperuricemia	CsA	Increased risk of developing hyperuricemia, increased endothelin-1 release, and reduced nitric oxide production.	Human	[137]
		Increased risk of developing hyperuricemia.	Human	[138]
	FK-506	Increased risk of developing hyperuricemia, increased endothelin-1 release, and reduced nitric oxide production.	Human	[137]
		Increased risk of developing hyperuricemia.	Human	[138]
		Lower incidence of hyperuricemia after FK-506 administration than after CsA.	R/M	[139]
	RAP	No effect on uric acid metabolism.	Human	[140]
	AZA	Good effect with pegloticase on decreasing blood urate concentration.	Human	[141]
	MMF	No effect or decrease in the mean uric acid concentration.	Human	[144]
	GCS	Elevation in blood urate levels.	Human	[145]

* CC—cell culture; ** R/M—review or meta-analysis article; *** N/A—not applicable. ATG—anti-thymocyte globulin; AZA—azathioprine; BAS—basiliximab; CsA—cyclosporin A; FK-506—tacrolimus, GCS—glucocorticoids; MMF—mycophenolate mofetil; RAP—sirolimus/rapamycin.

## Data Availability

Not applicable.
